# Clay‐associated microbial communities and their relevance for a nuclear waste repository in the Opalinus Clay rock formation

**DOI:** 10.1002/mbo3.1370

**Published:** 2023-07-10

**Authors:** Julia Mitzscherling, Steffi Genderjahn, Anja M. Schleicher, Alexander Bartholomäus, Jens Kallmeyer, Dirk Wagner

**Affiliations:** ^1^ GFZ German Research Centre for Geosciences, Section Geomicrobiology Potsdam Germany; ^2^ GFZ German Research Centre for Geosciences, Section Inorganic and Isotope Geochemistry Potsdam Germany; ^3^ Institute of Geosciences University of Potsdam Potsdam Germany

**Keywords:** deep biosphere, deep geological repository, microbial community, next‐generation sequencing, nuclear waste, Opalinus Clay

## Abstract

Microorganisms are known to be natural agents of biocorrosion and mineral transformation, thereby potentially affecting the safety of deep geological repositories used for high‐level nuclear waste storage. To better understand how resident microbial communities of the deep terrestrial biosphere may act on mineralogical and geochemical characteristics of insulating clays, we analyzed their structure and potential metabolic functions, as well as site‐specific mineralogy and element composition from the dedicated Mont Terri underground research laboratory, Switzerland. We found that the Opalinus Clay formation is mainly colonized by Alphaproteobacteria, Firmicutes, and Bacteroidota, which are known for corrosive biofilm formation. Potential iron‐reducing bacteria were predominant in comparison to methanogenic archaea and sulfate‐reducing bacteria. Despite microbial communities in Opalinus Clay being in majority homogenous, site‐specific mineralogy and geochemistry conditions have selected for subcommunities that display metabolic potential for mineral dissolution and transformation. Our findings indicate that the presence of a potentially low‐active mineral‐associated microbial community must be further studied to prevent effects on the repository's integrity over the long term.

## Graphical abstract

Biocorrosion and microbial mineral transformation can affect the safety of deep geological repositories for high‐level nuclear waste storage. We analyzed the composition and evaluated the importance of mineral‐associated microbial communities in Opalinus Clay for safety purposes. Rock‐attached communities were dominated by phyla capable of corrosive biofilm formation. Based on the abundance of potential iron‐reducing bacteria and available electron acceptors, iron reduction appeared to be the most important process. Site‐specific mineralogy and geochemistry have selected for subcommunities and specific metabolic functions.

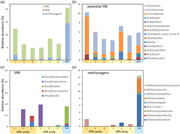

## 1 INTRODUCTION

Over their lifetime, European nuclear power plants are estimated to produce around 5.2 million m^3^ of nuclear waste, including spent nuclear fuel and waste generated during operation. Most countries have yet to develop and implement a safe long‐term waste management strategy (Harms, 2019). Deep geological repositories (DGRs) are the international consensus solution for safely isolating high‐level nuclear waste (HLW) from the biosphere. Repository layouts consist of multibarrier systems composed of technical barriers (engineered barriers) and geological barriers. The multibarrier design includes copper‐coated or iron steel spent fuel containers surrounded by a low‐permeability, swelling clay buffer (e.g., bentonite) within a stable host rock environment. Design concepts are, however, specific to each country depending on the national regulations and the local geology (International Atomic Energy Agency, 2003).

In several countries, such as France, Spain, Switzerland, Belgium, and Germany, clay formations have been selected as potential host rocks for the final disposal of HLW. Properties of clay formations, such as impermeability toward aqueous solutions, mechanical strength, extensive thickness, and homogeneity, are essential to their use as geological barriers (NAGRA, 2002). Due to their low hydraulic conductivity, high swelling capacity, plasticity, and sorption capacity for radionuclides, claystone is suitable for sealing off waste material. Over the past 30 years, the physical and chemical characteristics of potential clay host rocks, especially Opalinus Clay (OPA), have been studied extensively (Bossart et al., 2002; Mazurek, 1999; NAGRA, 2002; Pearson et al., 2003; Thury & Bossart, 1999). OPA is the preferred host rock for a DGR in Switzerland and is under consideration for Germany's DGR (BGE, 2020).

However, the diversity and functioning of microbial communities inhabiting this deep terrestrial subsurface environment remain poorly understood. Clay rocks have been demonstrated to suppress microbial growth (Stroes‐Gascoyne et al., 2010). The high swelling pressure (Keto, 2004) and low water activity due to small pore size and poor interconnectivity result in restricted availability of water (Brown, 1990) and nutrients (Courdouan et al., 2007) Hence, microbial activity is expected to be low or communities metabolically almost inactive (dormant) in clay‐rich environments (Stroes‐Gascoyne et al., 2007). But despite the inhospitable conditions, microorganisms are naturally present and have been detected in clay formations (Boivin‐Jahns et al., 1996; Lopez‐Fernandez et al., 2015). Microbial activity in a repository is predicted to occur in less densely packed interfacial environments and areas of disturbance, such as fractures and faults, where the geological barrier is disrupted. Excavation of a repository and placement of radioactive waste will provide more space and nutrient supply, such that the physical conditions necessary to suppress microbial activity may not be met (Stroes‐Gascoyne et al., 2010).

Microorganisms drive metabolic processes that can either be adverse or beneficial for the safety of a repository. Biofilm formation is an important issue predominantly at interfaces in a repository as it can facilitate clay mineral reduction and dissolution as well as microbially influenced corrosion (MIC) (Meleshyn, 2014). The reduction and dissolution of clay minerals related to the activity of iron‐reducing bacteria (IRB) can reduce the effectiveness of clay‐based technical and/or geological barriers (Leupin et al., 2017). Biofilm structures can further modify the interface between metal and solution, thereby accelerating the corrosion of metal surfaces such as waste containers. Given that a final repository should be stable over geological time, corrosion is of concern, although under anoxic conditions, it is usually a very slow process. MIC is mediated by a variety of bacterial and archaeal domains, but key contributors are sulfate‐reducing bacteria (SRB) due to their ability to induce pitting corrosion. Microbial fermentation of organic compounds, in addition to the abiotic corrosion of metals, can produce large amounts of gas (H_2,_ CO_2_) that could increase the gas pressure causing damage to the structural integrity of the repository and the surrounding rock. On the other hand, processes such as microbial sulfate reduction or hydrogenotrophic methanogenesis could reduce the gas pressure build‐up (Bagnoud, Chourey, et al., 2016; Meleshyn, 2014).

In OPA, evidence for the presence of living microorganisms includes phospholipid fatty acids, which estimated cell counts of 10^6^ living cells g^−1^ of dry claystone (Poulain et al., 2008; Stroes‐Gascoyne et al., 2007), as well as enrichment and cultivation of aerobic and anaerobic heterotrophs (Stroes‐Gascoyne et al., 2007). Although cultivation provides an insight into the viable and potentially active microbial community (Bagnoud et al., 2015; Poulain et al., 2008), isolates can reflect only a small fraction of the entire viable community. Isolation conditions select for specific microbial traits, while other organisms may be viable but not culturable (Oliver, 2005). Further approaches to characterize the microbial community composition of OPA included DNA‐based community analyses of water collected from boreholes (Bagnoud, de Bruijn, et al., 2016; Boylan et al., 2019). The in situ characterization of the clay rock‐attached microbial communities via DNA‐based analysis remains a challenge. Several attempts to extract, amplify, and sequence microbial DNA from the claystone directly were not or only partially successful. A multilaboratory microbial analysis of an OPA core involving laboratories from Switzerland, Germany, Japan, and France failed to extract amplifiable DNA (Stroes‐Gascoyne et al., 2007). Likewise, different DNA extraction approaches performed by Poulain et al. (2008) remained unsuccessful. In the study by Moll et al. (2013), microbial DNA could be isolated from unperturbed OPA for the first time. Another multilaboratory approach involving four laboratories from France, Switzerland, Germany, and Canada was successful in isolating and sequencing DNA from part of the samples recovered from a drill core (Bagnoud et al., 2015). However, despite using the same protocol, the results of the four laboratories showed only limited overlap. The most similar microbial communities were obtained from samples extracted in the same laboratory (Bagnoud et al., 2015; Moll et al., 2013), suggesting contamination during DNA extraction (Bagnoud et al., 2015). The most recent study generated inconsistent replicate 16S ribosomal RNA (rRNA) gene profiles that were associated primarily with contaminant sequences, again suggesting that the microbial profiles detected were not sample‐specific (Beaver et al., 2022).

Microbial communities and their metabolic functions inherent to natural ecosystems are complex. While samples from deep subsurface fluids have revealed abundant and diverse microbial life, limited work has described the corresponding biofilms on rock fracture and pore space surfaces (Casar et al., 2020). The aforementioned approaches, such as cultivation or community analyses of borehole water under different experimental conditions, tend to provide only a reductionist view of the microbial communities of this deep terrestrial subsurface, as they can solely account for parts of the total microbial community.

Environmental factors such as the physicochemical properties of the habitat usually represent constraints to microbial community assembly and functioning. According to the concept of the “mineralosphere,” minerals favor the development of specific microbial communities according to their mineralogy, nutritive content, and weatherability (Uroz et al., 2015). They can further drive significant differences in biodiversity between fluid and rock‐attached communities, as the host rock mineralogy is an important ecological driver in deep continental biospheres (Casar et al., 2020). In pristine subsurface environments, there is a characteristic sequence of microbial reduction processes based on the availability of key terminal electron acceptors and donors with nitrate dominating over Fe(III), sulfate, and CO_2_ when O_2_ is not present (Chapelle, 2000; Meleshyn, 2014). Since nitrate is not measurable in OPA rock (Pearson et al., 2003), Fe(III) from the clay minerals can serve as the primary electron acceptor in this environment (Yong et al., 2022). Thus, Fe(III)‐reducing microorganisms can be expected to dominate and outcompete SRB, which in turn would outcompete methanogenic organisms for electron donors, thereby inhibiting sulfate reduction and/or methane production (Chapelle, 2000). As this would contradict the results obtained from borehole water, there is a necessity for a protocol suited to investigate the rock‐attached microbial communities and their functions.

By applying different measures for contamination control during drilling and sample preparation, our study aimed for a comprehensive scientific basis to describe the OPA rock‐attached microbial community and evaluate the possible impact of microbial communities on the safety of a repository. We combined modern molecular biological methods such as high‐throughput sequencing with X‐ray diffractometry (XRD) and element analysis (inductively coupled plasma atomic emission spectroscopy) to explore the microbial community composition of OPA in the context of its physicochemical constraints.

## 2 MATERIALS AND METHODS

### 2.1 Drilling and sampling

#### 2.1.1 Sample site

**Figure 1:**
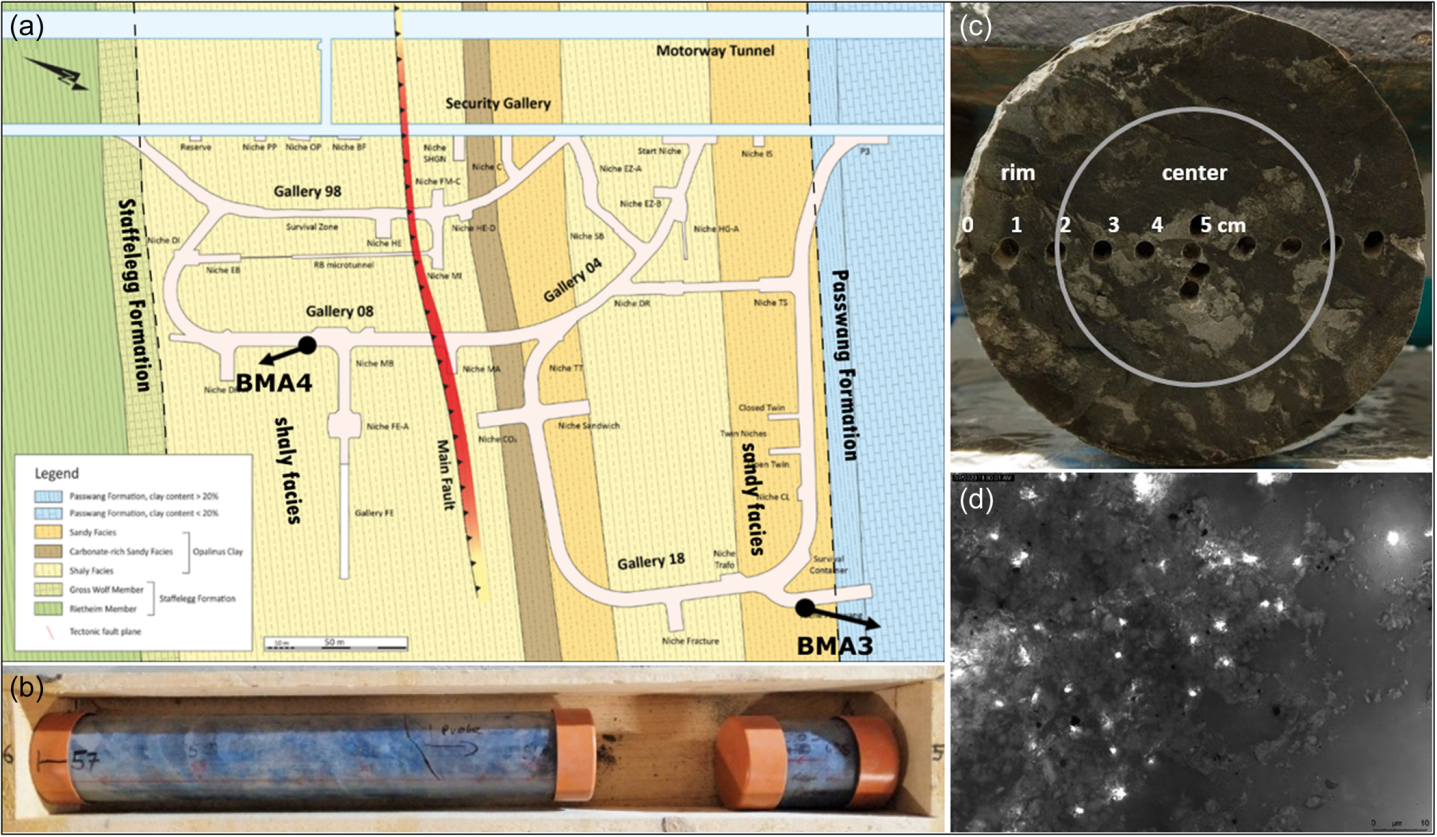
Drilling and contamination control. (a) Location of the BMA‐3 and BMA‐4 boreholes at the URL Mont Terri. BMA‐4 had a descending orientation, while BMA‐3 had an ascending orientation. Modified from Swisstopo (Bossart & Thury, 2008). (b) Drill cores with the outside colored by particle tracer solution. (c) Example of the contamination control from exterior to interior. Contamination entered the core less than 2 cm. (d) Fluorescing tracer particles under the microscope.

Two drill cores from the sandy and shaly facies of OPA as well as the Passwang formation (PW), a limestone stratigraphically located above the OPA formation, were recovered from the boreholes BMA‐3 and BMA‐4 at Mont Terri underground research laboratory (St‐Ursanne, Switzerland). The 15 m long core BMA‐3 was drilled in niche 5 of gallery 18 (Figure 1a). The core included 10 m of sandy facies as well as around 5 m of the adjacent PW formation. The 10 m long core BMA‐4 was drilled in Gallery 08 and retrieved from the shaly facies of OPA.

OPA is a sedimentary rock that formed around 180 million years ago. It is characterized by a very small pore size with a mean of 10–20 nm and a very low hydraulic conductivity of <10^−13^ m s^−1^ (Mazurek, 1999; Thury & Bossart, 1999). Redox conditions are reducing due to the presence of unoxidized pyrite and siderite, while the pH conditions are near‐neutral (NAGRA, 2002). OPA contains up to 1.5% (wt/wt) organic matter which is mainly associated with the solid phase (Pearson et al., 2003).

#### 2.1.2 Drilling procedure

To maintain anoxic conditions, drilling was performed with compressed nitrogen gas as previously described by Stroes‐Gascoyne et al. (2007). The triple core drilling technique provided 1.5 m long core sections with a diameter of 103.5 mm in a plastic liner. Plastic liners and the drill bit were cleaned with 70% ethanol before sampling and all the equipment was handled with sterilized gloves. For microbiological analyses, the cores within the plastic liners were sectioned immediately after drilling into 25 cm core sections using a circular saw. Core sections were quickly placed in a tubular foil (Gruber‐Folien GmbH & Co., KG), flushed several times with N_2_ gas, and sealed air‐tight. Core sections were stored at 4°C until further processing.

#### 2.1.3 Contamination assessment

To assess possible contaminations during the drilling procedure (e.g., from pressurized N_2_ gas or debris), we applied a contamination control using a fluorescent particle tracer similar to Stroes‐Gascoyne et al. (2007). As a tracer, we used horizon blue (SPL‐19N) fine‐grind fluorescent pigment dispersion (Radiant Color NV). Horizon blue fluorescent pigment particles have a size of 0.25–0.45 µm, a concentration of 1 × 10^12^ particles mL^−1^, and a green fluorescence under blue excitation (Friese et al., 2017). The tracer suspension was diluted 1:5 and introduced during drilling. Before drilling each 1.5 m core section, 10 mL of tracer solution was placed in a latex bag and attached to the drill bit. After introducing the core barrel and when starting the drilling, the impinging surface materials or the entering drill core split the latex bag. The tracer particle suspension was discharged in the vicinity of the drill bit and dispensed by the gas flow around the drill core (Figure 1b). This particular method of introducing particle tracers ahead of the drill bit has been widely used to assess microbial contamination (Colwell et al., 1992; Smith et al., 2000; House et al., 2003). In the laboratory, contamination was evaluated by sampling a subsample of the core sections from the exterior to the interior with an ethanol‐cleaned microdrill. Duplicate samples were collected at intervals of 1 cm from the rim to the center of the core (Figure 1c). A total of 50 mg sample material was weighed and suspended in 1 mL MilliQ water, shaken horizontally for 30 min, and allowed to settle for 5 min. About 10 µL aliquots were dispensed on a polycarbonate filter membrane and up to 100 randomly selected fields per sample were examined for the presence of fluorescent tracer particles (Figure 1d) on a Leica DM2000 fluorescence microscope using nonfluorescent immersion oil (Leica type F oil), a ×100 objective (Leica Plan Apo), and a blue filter set (Leica Filter Cube FI/RH; excitation BP 490/15 nm; emission BP 525/20 nm). Only the inner part of the core, where no tracer particles were detected, was sampled for downstream analyses.

#### 2.1.4 Sampling

Subsampling and sample preparation for microbiological purposes were performed in an N_2_ atmosphere of an anaerobic chamber. Core sections were removed from the liners and split into slices with a hammer and ethanol‐sterilized chisel. Per core section, around 100 g of clay from the interior of the core was recovered. Using an ethanol‐cleaned drill bit and an electric drill, the center of the core slices was perforated, and the resulting clay powder was collected in autoclave‐sterilized Nalgene® jars with screw caps. Drilling was performed slowly to avoid heating the sample. Only slices from inside the core sections were sampled as they were not in contact with the circular saw and air. Containers with rock powder were stored at −20°C until DNA extraction. Subsampling for geochemical and mineralogical analyses was done after the subsampling for microbial purposes. Rock chips were obtained by splitting pieces of the core slices with a hammer and chisel. They were powdered using an agate disk mill and sieved to obtain grain‐size fractions of <62 µm. This grain size was used for X‐ray fluorescence (XRF), total organic carbon (TOC), and total carbon (TC) analyses. Some of the powder was further homogenized with a micronizing mill to a grain size of <10 µm. This grain size was used for quantitative XRD analysis.

### 2.2 Mineralogy and geochemistry

#### 2.2.1 Mineralogy

The mineralogical composition was analyzed from eight samples each of the sandy and of the shaly OPA facies and from one sample of the PW formation. Samples of the sandy facies (BMA‐3) were obtained between 3.45 and 9.30 m from the gallery wall, while samples of the shaly facies (BMA‐4) were obtained between 3.97 and 9.13 m from the gallery. A PANalytical Empyrean XRD was used for the measurements, operating at 40 kV and 40 mA with Cu‐Kα radiation and a step size of 0.013°2*θ* from 4.6° to 85°. The mineralogy was first determined with the software EVA (version 11.0.0.3) by Bruker. Rietveld refinement for quantitative analysis was performed using the program BGMN and the graphical user interface Profex (version 3.10.2, Döbelin & Kleeberg, 2015). The error is expected to be in the range of 3 wt%.

#### 2.2.2 Geochemistry and elemental composition

Major elements and some minor elements were analyzed on fused beads using an AXIOS advanced XRF spectrometer (Malvern PANalytical). The quantification level for the wt% range is 0.02 wt% and 10 ppm for minor elements. The analysis resulted in an abundance of oxides which were later transformed into element concentrations. H and C were analyzed using a Euro EA Elemental Analyzer. H_2_O and CO_2_ were calculated. Additionally, sulfur was analyzed using an Eltra CS 2000 instrument, and nitrogen using a Euro EA 3000 elemental analyzer.

To determine anion concentrations, sample material was leached according to Blume et al. (2011). Five grams of sample material was suspended in 25 mL of MilliQ water and horizontally shaken for 90 min at 120 rpm. The sample material was sedimented by centrifugation for 15 min at 10,000*g* and the supernatant was filtered through a 0.22 µm cellulose acetate filter (Minisart High Flow; Sartorius). Anions were analyzed using a Sykam S 155 ion chromatograph with an integrated suppressor. As a mobile phase, we used an eluent containing 636 mg/L Na_2_CO_3_ and 7.5 mg/L NaSCN with a flow rate of 1 mL/min. A Sykam SykroGel Ax300 AB‐A01 column suitable for quantification of anions F^−^, Cl^−^, Br^−^, NO_2_^−^, NO_3_^−^ SO_4_^2−^, and PO_4_^3−^ was used at a temperature of 60°C. Triplicates of 50 µL leachate and several certified multielement standards were injected using a Sykam S 5300 sample injector. Concentrations were determined via peak area evaluations.

#### 2.2.3 TC and TOC

A mass spectrometer DELTA V Advantage from Thermo Fisher Scientific and the Element analyzer EA Isolink were used for TC and TOC analyses. For the TC sample preparation, ca. 5 mg material was analyzed in Sn‐capsules with an analytical precision of TC < 0.1%. For TOC, 5 mg material was weighed in Ag‐capsules, in situ acid treated using first 3% and second 20% HCl, and analyzed with an analytical precision of TOC < 0.2%. The difference between TC and TOC results in total inorganic carbon (TIC) contents. With the TIC contents, the calcite contents were calculated using the relative atom masses.

### 2.3 Microbial community analysis

#### 2.3.1 DNA extraction

DNA was extracted from three biological replicates of each OPA facies and one sample from the PW. Replicates were obtained from different depth locations of the drill cores: samples representing the sandy facies were obtained from core BMA‐3 3.85, 5.70, and 7.25 m from the gallery wall and are referred to as OPA sandy‐1 to OPA sandy‐3. The sample from the PW formation (PW) was obtained from the same core at 10.75 m from the gallery wall. The samples OPA shaly‐1 to OPA shaly‐3 were sampled from core BMA‐4 4.26, 6.46, and 8.90 m from the gallery.

Total genomic DNA was extracted using the DNeasy PowerSoil Pro Kit (Qiagen). The lysate of five extraction replicates was pooled on one MB spin column to enhance the yield of DNA. In addition to the samples, DNA was extracted from different negative controls (NCs): a blank kit extraction without adding any sample (kit NC) and from Sterivex^TM^ filters (Merck Millipore) used to filter the N_2_ gas from the anaerobic chamber before sample preparation (inflow) and afterward (outflow).

#### 2.3.2 Library preparation and sequencing

Recovered DNA was amplified using the primer pair 515F and 806R (Caporaso et al., 2011) targeting the hypervariable region V4 of the 16S rRNA gene. A blank PCR reaction without template DNA was performed as an additional NC. To obtain enough PCR product, three reactions from the same template were pooled and purified using the AMPure® XP beads (Beckman Coulter) and quantified using a Qubit Fluorometer 2.0 (Life Technologies). Illumina paired‐end sequencing was performed at Eurofins Genomics on an Illumina MiSeq machine with MiSeq V3 chemistry (2 × 300 bp paired‐end reads). For each genomic DNA sample, two technical replicates with different barcodes were amplified by PCR and sequenced.

The sequencing library was demultiplexed using cutadaptv3.4 (Martin, 2011) using the following parameters: ‐e 0.2 ‐q 15,15 ‐m 150 ‐‐discard‐untrimmed. The amplicon sequence variants (ASVs) were generated using trimmed reads and the DADA2 package v1.20 (Callahan et al., 2016) with R v4.1 using the pooled approach with the following parameters: truncLen=c(240,200), maxN=0, rm.phix=TRUE, minLen=200. Taxonomic assignment was carried out using DADA2 and the SILVA database v138 (Quast et al., 2012). Subsequently, ASVs representing chloroplasts, mitochondria, and singletons were removed using a custom R script.

#### 2.3.3 Statistics and data visualization

**Figure A1:**
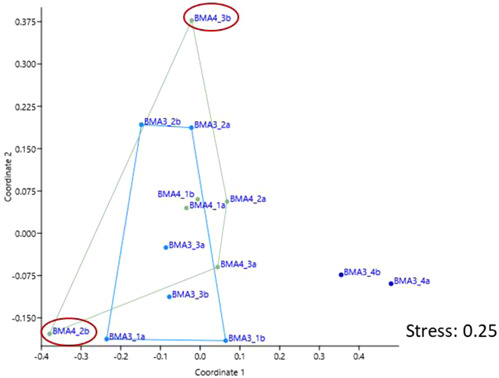
NMDS plot of duplicate samples to evaluate the similarity. The samples BMA4_3b and BMA4_2b clustered further apart and were thus excluded from the analysis. NMDS, nonmetric multidimensional scaling.

Before statistical analysis, ASVs occurring in the NCs with >20 reads were removed from the library. Absolute read counts were transformed into relative abundances. The similarity of duplicate samples was evaluated visually in a nonmetric multidimensional scaling (NMDS) plot before duplicates were merged for the microbial community analysis (Figure A1). For statistical purposes, mineralogical and geochemical data were normalized using *z*‐score transformation and we used the mean of the given range of *i*–*s* values (Supporting Information: Data S1). Analysis of the microbial community and its relation to the environmental parameters was assessed using the Past 4.09 software (Hammer et al., 2001) and R, especially the packages vegan, MASS, igraph, RColorBrewer, Hmisc, Matrix, and gplots. A principal component analysis (PCA) based on Euclidean distance was used to assess variation of the mineralogy across the two different OPA facies and the PW formation. Beta diversity of the microbial communities was assessed in an NMDS ordination plot using the Bray–Curtis dissimilarity. Environmental factors influencing the community composition were determined by an environmental fit into the ordination. The significance of the variance introduced by the identified environmental factors was tested using Mantel tests and confirmed by a permutational approach as implemented in the adonis function of the vegan package. Associations of microbial phyla and classes with the rock mineral composition were evaluated by calculating the Pearson correlation coefficient. The occurrence of specialist taxa was detected by an indicator species analysis (indicator value > 0.8, *p* < 0.05) using the R package labdsv implementing the algorithm of Dufrene and Legendre (1997). The co‐occurrence network was built on Pearson correlation based on ASVs with a summed relative abundance of >1% across all samples and a correlation coefficient >0.6 resulting in an undirected network with scale‐free topology. Network construction and visualization were carried out with R using the packages igraph and RColorBrewer. Network modules were detected using random walk (function cluster_walktrap, igraph package), and the layout was calculated using the Fruchterman–Reingold algorithm (function layout.fruchterman.reingold, igraph package). To obtain the final correlation of environmental parameters with the modules, the following procedure was applied. First, all ASVs were correlated to all environmental parameters. Second, for each environmental parameter, the mean of the correlations of the ASVs in a module was taken. Thus, the correlation of an environmental parameter and a module only occurred strongly if most of the ASVs of the module showed the same correlation. The heatmap was generated using the gplot R package.

## 3 RESULTS

### 3.1 Mineralogy and geochemical properties of the rock

**Figure 2:**
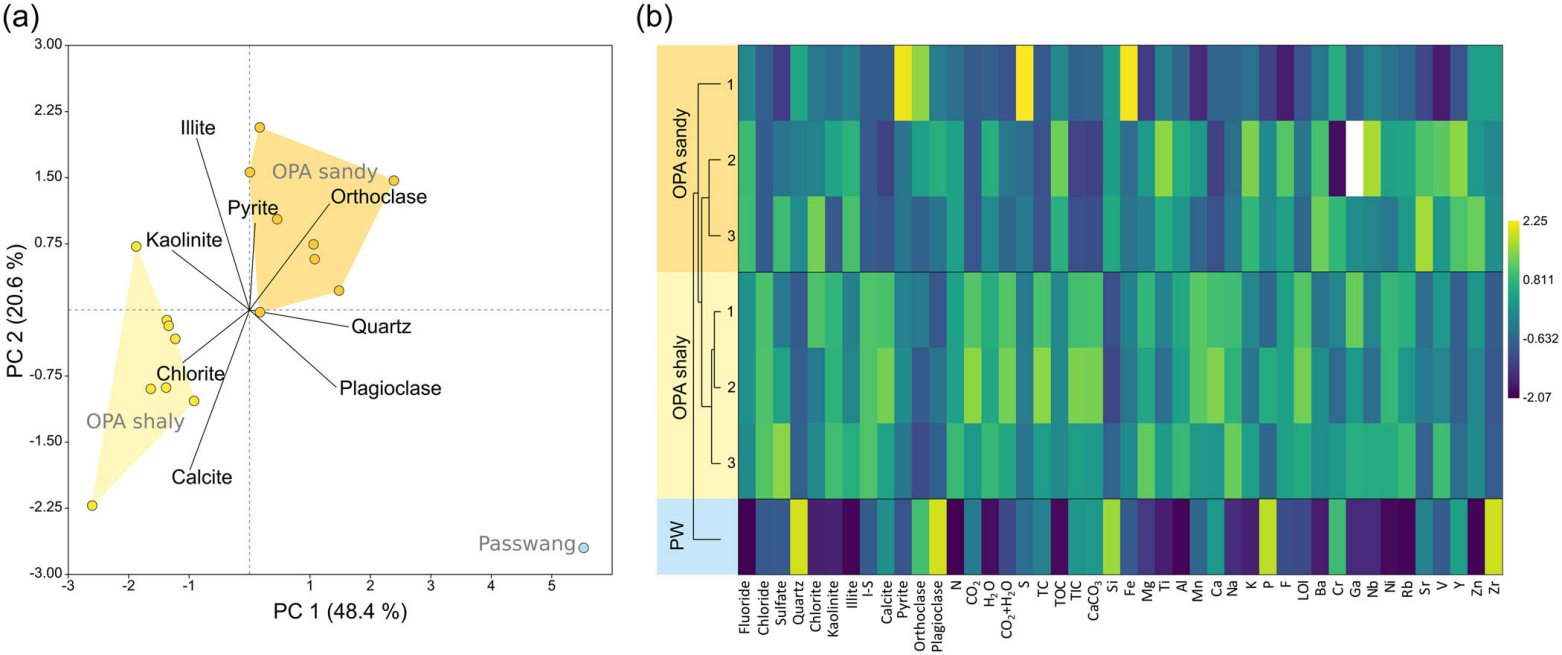
Mineralogy of the sandy and shaly OPA facies and the PW. (a) Principal component analysis of the mineral composition of eight samples per clay facies and the Passwang sample. Light yellow represents samples of the shaly facies (OPA shaly), yellow ochre of the sandy facies (OPA sandy), and blue represents the PW. (b) Geochemical and mineralogical parameters of samples used for the microbial community analysis. Data were *z*‐score transformed. The blank box represents missing data. Absolute numbers can be found in Supporting Information: Data S1. I–S, mixed‐layer illite–smectite; LOI, loss of ignition; OPA, Opalinus Clay; PW, Passwang formation.

The stratigraphy of the OPA and overlying PW was composed of quartz, clay minerals (chlorite, kaolinite, illite, and illite–smectite), calcite, pyrite, and feldspar (orthoclase and plagioclase) at different concentrations (Supporting Information: Data S1). A PCA (Figure 2a) of the minerals showed a clear separation between the two OPA facies (sandy and shaly) and the PW sample along the *x*‐axis, which explained 48.4% of the variance. The separation between OPA and PW was mainly caused by the high abundance of quartz and plagioclase in the PW formation and higher abundances of illite and kaolinite in the OPA samples (Figure 2a,b). Samples of the two different OPA facies were separated along the *y*‐axis, which explained 20.6% of the variance. The difference between both facies was caused by a higher calcite content in the OPA shaly facies and higher quartz and orthoclase contents in the OPA sandy facies.

To further describe the environmental conditions of the seven samples used for microbial community analysis, we determined anion concentrations, water (H_2_O), carbon (TOC, TIC, and TC), and calcium carbonate (CaCO_3_) content as well as the concentrations of different elements including nitrogen (N) and sulfur (S) (Figure 2b and Supporting Information: Data S1). Results confirmed the difference of the PW formation from the OPA. In addition to a higher quartz and plagioclase concentration, the PW formation was characterized by higher amounts of Si, P, and Zr, while the fluoride concentration, the N, H_2_O, TOC, Al, K, Ni, Rb, and Zn contents were the lowest. In general, the two OPA facies were similar compared to PW. However, the OPA shaly facies was characterized by higher contents of chloride, CO_2_, TC, TIC, CaCO_3_, Mg, Mn, Ca, Na, and Ni compared to the OPA sandy facies. Within the OPA sandy facies, the sample located closest to the gallery wall (OPA sandy‐1) was characterized by the highest pyrite (FeS_2_) content and concomitantly the highest sulfur and iron contents.

### 3.2 Microbial community structure and composition

**Figure 3:**
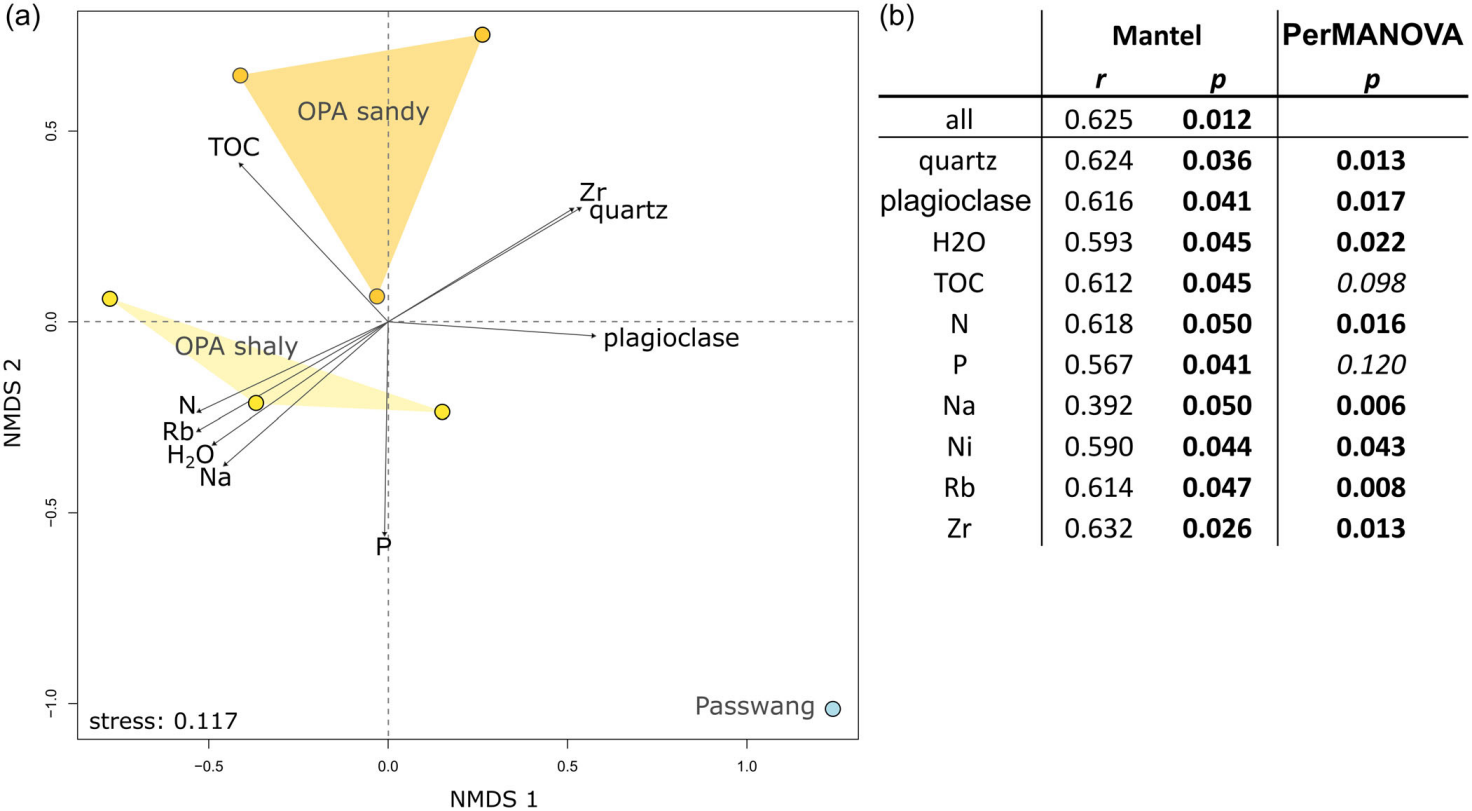
Structure of the microbial communities in OPA and the Passwang formation, and their relation to environmental parameters. (a) Nonmetric multidimensional scaling plot based on 1675 ASVs obtained from OPA and the Passwang formation. Environmental factors that contribute significantly to the variance of the community data are shown. Each sample point was generated using the mean relative ASV abundances from two technical replicates. (b) Environmental factors determined by the Mantel test and PerMANOVA that contribute significantly (*p* < 0.05) to the variance of the community data. Significant *p*‐values are bold. ASV, amplicon sequence variant; MANOVA, multivariate analysis of variance; NMDS, nonmetric multidimensional scaling; OPA, Opalinus Clay.

Similar to mineralogy, the microbial communities of the OPA sandy and OPA shaly facies formed clusters well apart from the community of the PW formation (Figure 3a). According to the Mantel test (Figure 3b), the cumulative environmental factors were strongly correlated with the microbial community. Single parameters explaining the variance of the microbial community structure included the minerals quartz and plagioclase as well as H_2_O, TOC, N, P, Na, Ni, Rb, and Zr. Along the *x*‐axis of the ordination plot, the variance of the microbial communities was correlated mainly with plagioclase and partly with quartz and Zr, which were enriched in the PW sample (Figure 3a). Furthermore, OPA samples correlated with TOC, which was more highly concentrated in the OPA samples (0.8%–1.0%) compared to the PW sample (0.5%). The separation of OPA samples from the PW sample along the *y*‐axis was mainly explained by P, which was enriched in the PW sample. The variance of the OPA sandy and OPA shaly microbial communities along the *y*‐axis correlated with slightly higher N, Rb, Na, and H_2_O contents in the OPA shaly facies as well as with higher quartz and Zr contents in the OPA sandy facies.

**Figure 4:**
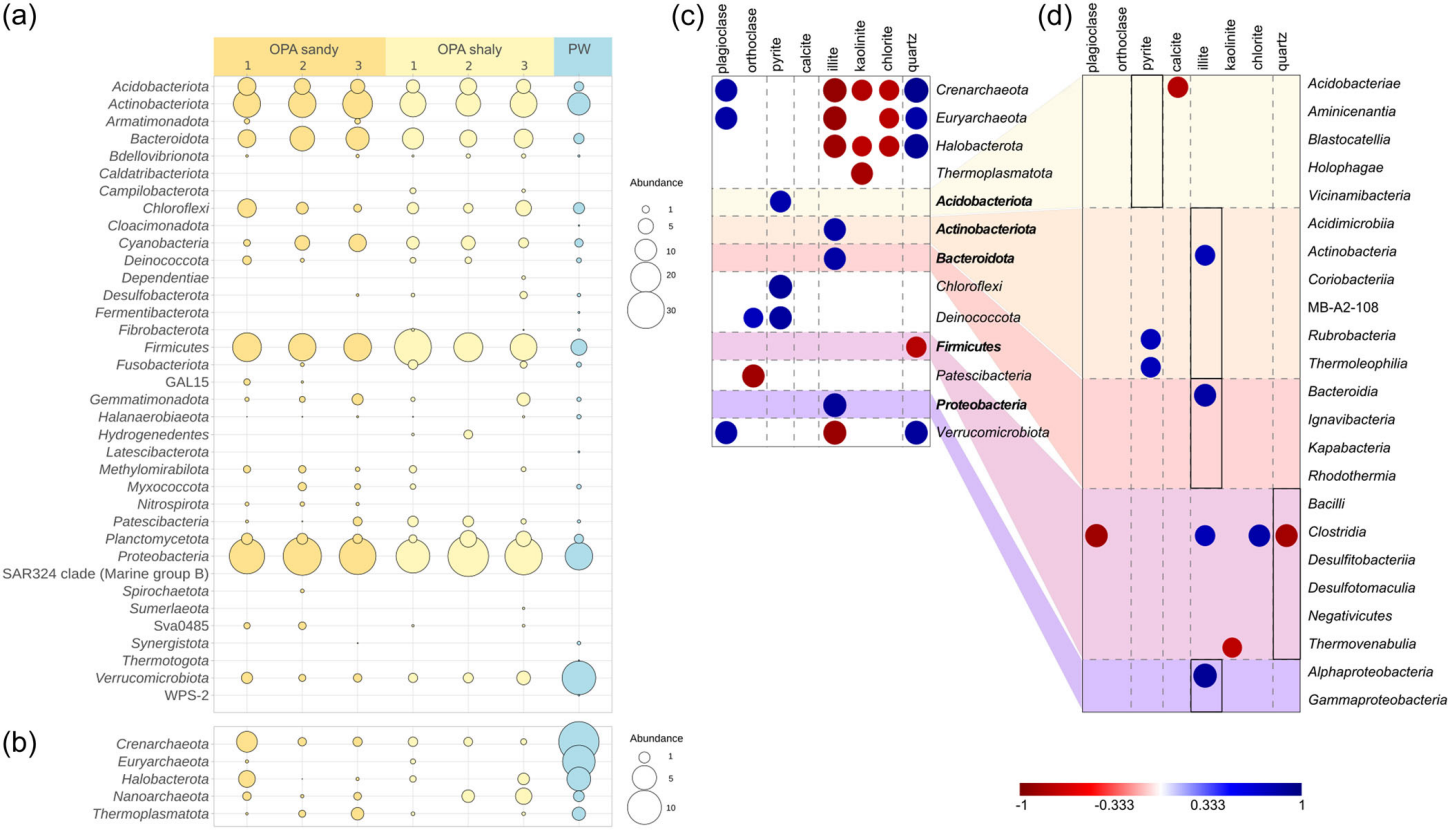
Microbial community composition and correlation to mineralogy. (a) Relative abundance of bacterial phyla and (b) of archaeal phyla in %. (c) Pearson correlation of minerals with archaeal and bacterial phyla. The most abundant bacterial phyla of Opalinus Clay are bold. (d) Correlations of bacterial classes of the most abundant phyla in Opalinus Clay. Only significant correlations (*p* < 0.05) are shown. Bubble size indicates the significance (bigger bubbles represent lower *p*‐values) while the color indicates a positive (blue) or negative (red) correlation (*r*‐value).

Microbial communities of both OPA facies were strongly dominated by bacteria with a relative abundance ranging from 93.3% to 99.0%. In contrast, bacteria in the adjacent PW formation comprised only 69% of the total microbial community, while 31% belonged to Archaea (Table A1). In total, ASVs belonged to 36 bacterial and 5 archaeal phyla. Bacteria in OPA were dominated by the phyla Proteobacteria, Firmicutes, Actinobacteriota, Bacteroidota, and Acidobacteriota (Figure 4a). These phyla were also amongst the dominant phyla in the PW formation but were less abundant compared to OPA samples. Phyla predominating in the PW formation were different from OPA and included Verrucomicrobiota as well as the archaeal phyla Crenarchaeota, Euryarchaeota, and Halobacterota (Figure 4b).

**Figure A2:**
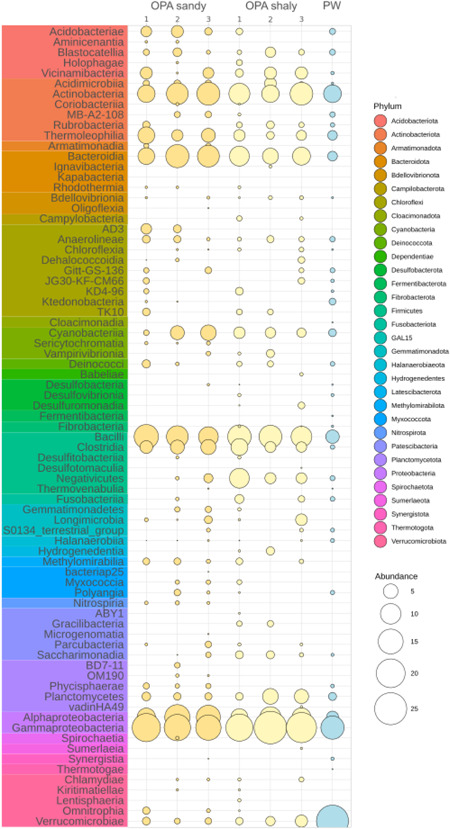
Relative abundances of bacterial classes. Classes are colored according to their phylum affiliation.

**Figure A3:**
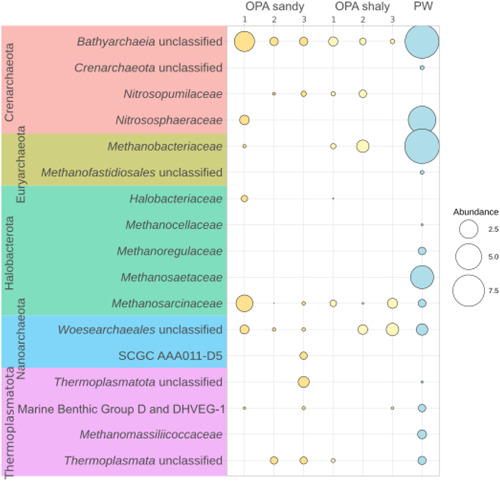
Relative abundances of archaeal families. Families are colored according to their phylum affiliation.

Highly abundant classes of the dominant bacterial phyla were the Acidobacteriae within the shaly facies and Vicinamibacteria in both facies, Actinobacteria and Thermoleophilia, Bacteroidia, Bacilli and Clostridia in both clay facies, as well as Negativicutes in the OPA sandy facies. Alphaproteobacteria and Gammaproteobacteria were found to be abundant in both clay facies (Figure A2). Verrucomicrobiae were the most abundant Verrucomicrobiota in the PW formation. Here, Nitrosphaeraceae, unclassified Bathyarchaeia, Methanobacteriaceae, and Methanosaetaceae were the most prominent families among archaeal taxa (Figure A3). In addition to the abundant phyla and classes, the PW formation comprised methanogenic archaea that were not found in the clay samples, such as unclassified Methanofastidiosales, Methanocellaceae, Methanoregulaceae, and Methanomassiliicoccaceae.

An indicator species analysis on the ASV level revealed the occurrence of unique taxa in the PW formation (Table A2). We found 10 indicator ASVs, of which five were affiliated with bacteria and five with archaea. The archaeal ASVs comprised unclassified Bathyarchaeia (2 ASVs), Nitrosphaeraceae (1), *Methanobacterium* (1), and *Methanosaeta* (1). Bacterial ASVs were affiliated with *Nocardioides* (1), *Rhodoferax* (1), *Luteolibacter* (2), and unclassified Verrucomicrobiaceae (1). The 10 indicator ASVs represented about 40% of the entire community of the PW formation. *Luteolibacter* ASVs had an abundance of 17.6%, while ASVs affiliated with the methanogens *Methanobacterium* and *Methanosaeta* made up 9% of the total community.

### 3.3 Mineral‐associated microbial community in OPA

Archaeal phyla such as Crenarchaeota, Euryarchaeota, and Halobacterota and the bacterial phylum Verrucomicrobiota mainly occurring in the PW formation showed a significant positive correlation with the main components of the PW formation: quartz (*r* = 0.94, *r* = 0.84, *r* = 0.92, and *r* = 0.88) and plagioclase (*r* = 0.86, *r* = 0.85, *r* = 0.75, and *r* = 0.85) (Figure 4c). A negative correlation of those phyla was observed with chlorite (*r* = −0.77, *r* = −0.77, *r* = −0.78, and *r* = −0.75), kaolinite (*r* = −0.80, *r* = −0.70, *r* = 0.78, and *r* = −0.74) and illite (*r* = −0.91, *r* = −0.90, *r* = −0.88, and *r* = −0.89) being less abundant in the PW formation. Illite, the main clay mineral in OPA, was positively correlated with some of the most abundant bacterial phyla in OPA such as Actinobacteria (*r* = 0.85), Bacteroidota (*r* = 0.85), and Proteobacteria (*r* = 0.90). Other abundant bacterial phyla in the OPA facies, such as Firmicutes, did not correlate with the clay minerals directly but showed an inverse correlation with quartz (*r* = −0.79). Acidobacteriota and the less abundant Chloroflexi as well as Deinococcota were positively correlated with pyrite (*r* = 0.81, *r* = 0.90, and *r* = 0.87), which was enriched in the sample OPA sandy‐1 (Figures 2 and 4).

**Figure 5:**
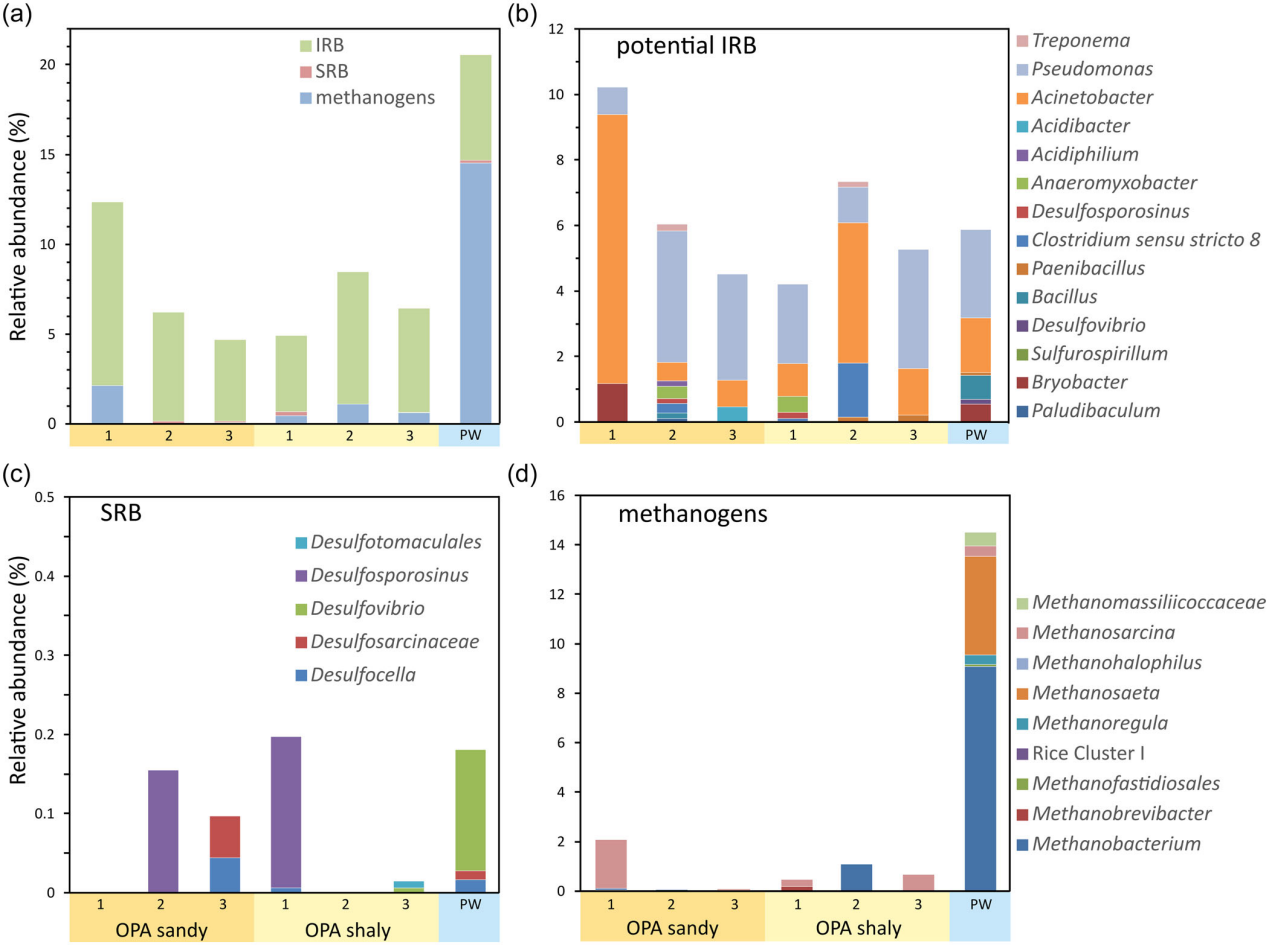
Cumulative relative abundances (a) of the different functional traits in Opalinus Clay and the Passwang formation. (b) Relative abundances of different genera of potential iron‐reducing bacteria (IRB), (c) sulfate‐reducing bacteria (SRB), and (d) methanogenic archaea. Genera of the different functional traits were selected manually based on their taxonomy.

Within the phylum Actinobacteroidota, the class Actinobacteria was most abundant and thus responsible for the correlation with illite (*r* = 0.78) (Figure 4d). Thermoleophilia, another abundant class within the phylum Actinobacteroidota and Rubrobacteria correlated with pyrite (*r* = 0.77). Within the phylum Bacteroidota, the class Bacteroidia was most abundant and responsible for the correlation with illite (*r* = 0.85). Clostridia were not the most abundant class of the Firmicutes but like the phylum, their abundance negatively correlated with quartz (*r* = −0.85). In addition, Clostridia showed a negative correlation with the mineral plagioclase (*r* = −0.87) and a positive correlation with the clay minerals illite (*r* = 0.78) and chlorite (*r* = 0.83). Although Alphaproteobacteria were less abundant than Gammaproteobacteria, they were responsible for the positive correlation of Proteobacteria with illite (*r* = 0.91).

Since microbial processes such as corrosion, mineral alteration, or gas pressure changes can affect the integrity of engineered and geological barriers in a final repository, we aimed at evaluating the potential for specific microbial processes in OPA. Therefore, we focused on the relative abundance of functional microbial traits including IRB, SRB, and methanogenic archaea (Figure 5). We found potential IRB throughout all OPA samples and in the PW formation with an abundance ranging from 4.3% to 10.2%. The highest abundance was observed in OPA sandy‐1, which possessed the highest pyrite content. Accordingly, the abundance of potential IRB was linearly correlated with pyrite (*p* = 0.018, Pearson's *r* = 0.84). Potential iron reducers were diverse with 73 ASVs belonging to the genera *Acinetobacter* (25 ASVs), *Acidiphilium* (3), *Acidibacter* (1), *Anaeromyxobacter* (4), *Bacillus*, *Bryobacter* (3) *Clostridium sensu stricto 1* (3) and *Clostridium sensu stricto 8* (1), *Desulfosporosinus* (1), *Desulfovibrio* (2), *Paenibacillus* (4), *Paludibaculum (1)*, *Pseudomonas* (17), *Sulfurospirillum* (1), *Treponema* (2), and *Vibrio* (1) (Bai et al., 2021; Herrera & Videla, 2009; Kulichevskaya et al., 2014; Pan et al., 2017; Figure 5b). *Acinetobacter* dominated in OPA sandy‐1 and OPA shaly‐2, where we observed the highest potential IRB abundance with 8.2% and 4.3%, respectively, while *Pseudomonas* dominated the remaining samples with abundances ranging between 2.4% and 4.0% relative abundance (Supporting Information: Data S2). SRB represented only a minority of sequences amplified from the OPA samples and the PW formation with abundances <0.2% (Figure 5c). In line with the indicator species analysis (Table A2), methanogenic archaea were found to be abundant in the PW formation, where they possessed the highest abundance of methanogens of all samples with 14.5% (Figure 5d). Within the OPA samples their abundance ranged from <1% to 2.1%. Like potential IRB, methanogenic archaea were quite diverse with 34 ASVs affiliated with *Methanobacterium* (10 ASVs), *Methanobrevibacter* (1), unclassified *Methanofastidiosa* (1), Rice Cluster I (1), *Methanoregula* (1), *Methanosaeta* (5), *Methanohalophilus* (1), *Methanosarcina* (12), and unclassified *Methanomassilliicoccaceae* (2). In OPA, methanogens were mainly affiliated with *Methanosarcina* and *Methanobacterium*. The PW sample was also dominated by *Methanobacterium* with about 9% relative abundance. *Methanosaeta* represented almost 4% of that sample. Due to a higher quartz and plagioclase content and a lower chlorite and illite content in the PW formation compared to OPA, methanogenic archaea were positively correlated with quartz (*p* = 0.007, *r* = 0.89) and plagioclase (*p* = 0.016, *r* = 0.85), while they were negatively correlated with chlorite (*p* = 0.034, *r* = −0.79) and illite (*p* = 0.004, *r* = −0.92).

### 3.4 Co‐occurrence network

To explore co‐occurrence patterns in the rock environments of OPA and the PW formation, a microbial association network was constructed that featured only significant correlation relationships for ASVs with a notable prevalence (>1% cumulative relative abundance across all samples). 156 out of 1675 ASVs were finally employed in the network analysis. The co‐occurrence was explored based on strong correlations (using Pearson's *r* > 0.6) and was constructed using the Walktrap community finding algorithm (Pons & Latapy, 2005).

**Figure 6:**
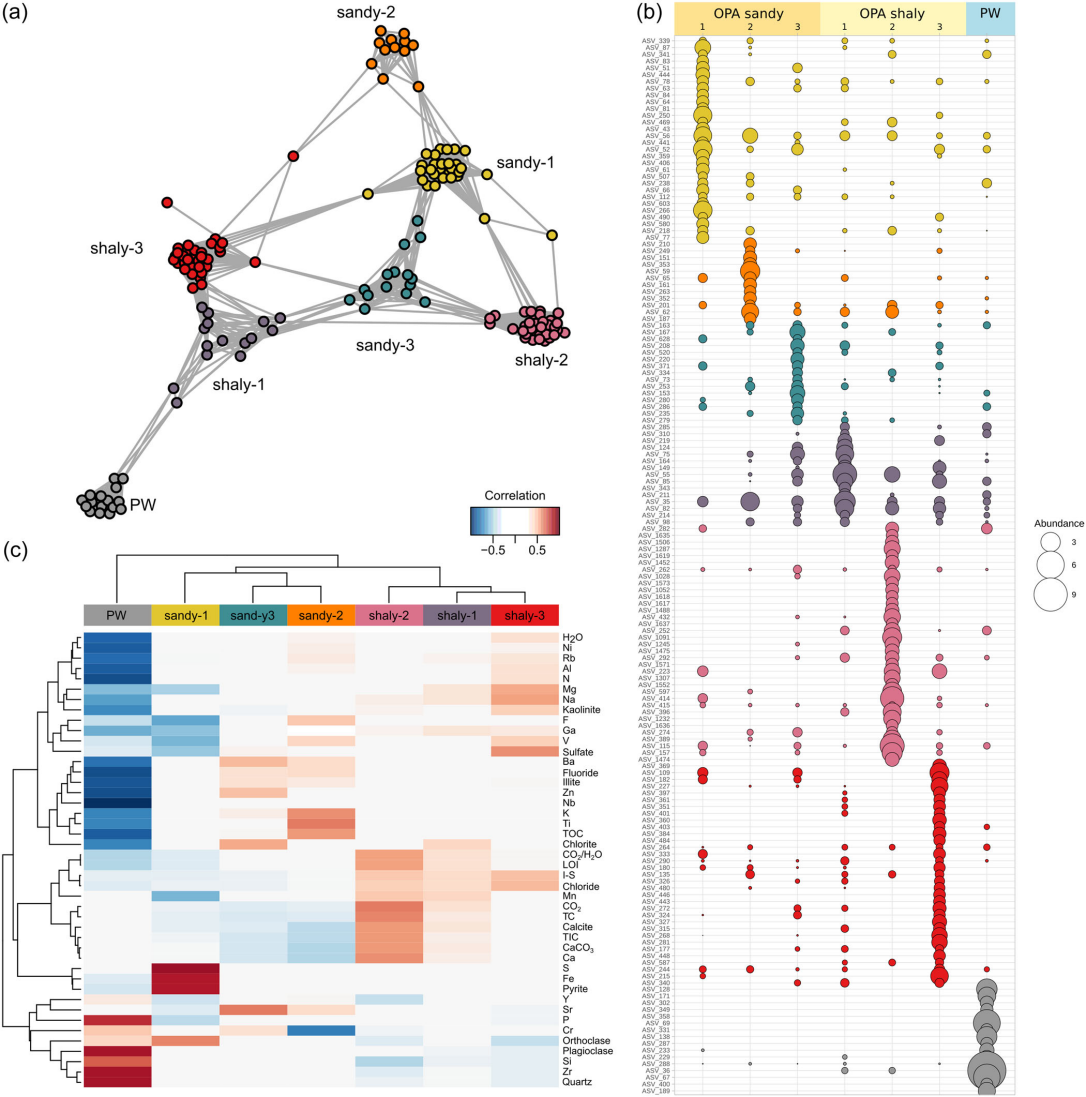
(a) Network of co‐occurring ASVs with >1% relative abundance across all samples based on correlation analysis. A connection represents a strong correlation (Pearson's *r* > 0.6). Nodes are colored by module classification. (b) Abundance of ASVs of the co‐occurrence network. ASVs are colored by the module they were assigned to. Taxonomic classification and relative abundance data can be found in Supporting Information: Data S3. (c) Correlation of modules with mineralogical and related geochemical parameters. Red, positive correlation; blue, negative correlation. ASV, amplicon sequence variant; OPA, Opalinus Clay; PW, Passwang formation.

The resulting network (Figure 6a) consisted of 156 nodes (i.e., ASVs) and 1837 edges with an average node connectivity of 23.5. Overall, the rock microbial network comprised highly connected ASVs (edge‐to‐node ratio 11.8) forming a clustered topology with densely connected groups of nodes (modules). The clustering coefficient (i.e., the degree to which they tend to cluster together) was 0.91 and the modularity index was 0.73. Modularity values > 0.4 indicate a modular structure of the network (Newman, 2006).

**Figure A4:**
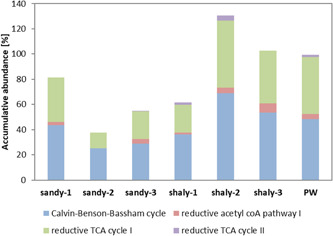
Abundance of carbon fixation pathways predicted with Picrust2 based on amplicon sequencing data.

The structural analysis showed that the cluster formation (modules) was in accordance with the number of rock samples analyzed. Each module had a unique composition of co‐occurring ASVs, which predominated only at one sample location (Figure 6b and Supporting Information: Data S3). For this reason, modules were named after the sample in which the co‐occurring ASVs predominated. Furthermore, the composition of co‐occurring microorganisms in each module was correlated with specific mineralogical and geochemical parameters being characteristic of the respective sample (Figure 6c). In detail, co‐occurring ASVs of module PW strongly correlated with quartz and plagioclase as well as with Si, P, and Zr which dominated the PW formation. Co‐occurring taxa of module OPA sandy‐1 strongly correlated with pyrite (FeS_2_) and thus with the Fe and S content, which were enriched in that sample (Figure 2b). Higher contents of CO_2_, TC, TIC, calcite, CaCO_3,_ and Ca characterizing sample OPA shaly‐2 correlated with co‐occurring taxa of module OPA shaly‐2 (Figure 6c), which included high abundances of carbon‐fixing *Planctomycetes* and Proteobacteria (Data S3). At the same time, predicted pathways for carbon fixation revealed by PICRUSt2 analysis (Douglas et al., 2020) were significantly enriched at this site (Figure A4). A higher TOC, Ti, and K content in OPA sandy‐2 was correlated with ASVs comprising module OPA sandy‐2. Co‐occurring ASVs of the modules OPA shaly‐1 and ‐3 as well as OPA sandy‐2 and ‐3 exclusively contained bacterial taxa (Supporting Information: Data S3). Module OPA sandy‐1 and OPA shaly‐2 contained three and one archaeal ASV, respectively, while in module PW the number of archaeal and bacterial ASVs was equal with seven ASVs each. Module PW was dominated by typical taxa of the PW formation including Bathyarchaeia (3.3%), *Methanobacterium* (5.9%), *Methanosaeta* (3.2%), and two ASVs affiliated with *Luteolibacter* (11.9% and 5.7%), that were also identified as indicator species. The remaining modules were not dominated by single ASVs. Most abundant ASVs (>2%) across all modules were affiliated with the phyla Acidobacteriota, Actinobacteria, Bacteroidota, Cyanobacteria, Alpha‐ and Gammaproteobacteria, Planctomycetota, and Verrucomicrobiota (Supporting Information: Data S3). Acidobacteriota were abundant in modules OPA shaly‐2 and shaly‐3, Cyanobacteria were enriched in modules OPA shaly‐1 and sandy‐2, while Firmicutes and Gammaproteobacteria were abundant in modules OPA shaly‐1 and ‐2 as well as OPA sandy‐1 and ‐2. In summary, the co‐occurrence network with its intrinsic structure revealed sample/site‐specific differences.

## 4 DISCUSSION

### 4.1 The OPA microbial community

The present study provides a comprehensive insight into OPA rock as a microbial habitat, the mineralogical constraints, as well as potential consequences on the safety of a repository. Different measures for contamination control were applied to guarantee the reliability of our results. Measures included the application of contamination tracers during drilling, sampling, and sequencing of the N_2_ gas atmosphere during sample preparation and different controls during DNA extraction and amplification. Hence, our study strongly expands the knowledge of the rock‐attached microbial communities in unperturbed OPA.

**Figure A5:**
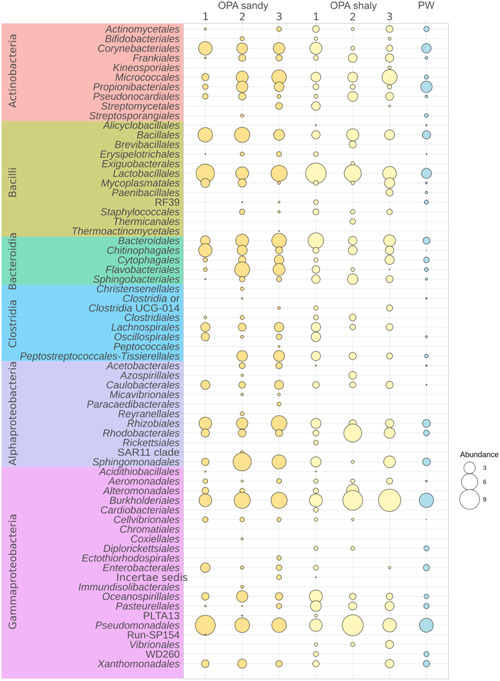
Relative abundance of bacterial orders of the dominant classes: Actinobacteria, Bacilli, Bacteroidia, Clostridia, Alpha‐, and Gammaproteobacteria.

Despite the harsh living conditions in OPA rock, we identified a diverse microbial community (Figures A2, A3, A5, and A7) with Gammaproteobacteria dominating over Alphaproteobacteria, followed by the phylum Firmicutes (Figures 4 and A1). The dominant phyla inherent to OPA were also identified by DNA‐based approaches in other natural clay formations such as bentonite (Beaver et al., 2022; Lopez‐Fernandez et al., 2015) and Boom Clay (Boivin‐Jahns et al., 1996), or by culture‐dependent approaches in bentonite (López‐Fernández et al., 2014).

We observed relatively homogenous microbial communities in both OPA facies (Figure 4a,b), thereby contrasting the results of Bagnoud et al. (2015) who concluded that the distribution of microorganisms in the rock is highly heterogeneous. In the study by Bagnoud et al. (2015), three laboratories were able to extract and sequence DNA from OPA, but the results had limited consistency. Depending on the analyzing laboratory, the described community was mainly composed of Beta‐ and Alphaproteobacteria as well as Bacteroidota or of Beta‐, Alpha, and Deltaproteobacteria, while the study by Moll et al. (2013) also revealed the dominance of Firmicutes. Furthermore, Bagnoud et al. (2015) found that archaeal sequences in the shaly OPA facies were dominated by Euryarchaeota, composed of Methanomicrobia and Methanobacteria. In our study, the small archaeal community in the shaly facies was dominated by Bathyarchaeia while *Methanobacterium* was very abundant within the PW formation.

While some taxa previously isolated from OPA like *Exiguobacterium*, *Bacillus*, *Microbacterium*, *Nocardioides*, *Alicyclobacillus* (Poulain et al., 2008), or *Sporomusa* (Moll et al., 2013) could not be identified by our DNA‐based approach, or only in single samples at low abundances. Other genera such as *Sphingomonas* (Poulain et al., 2008) were ubiquitously abundant.

Following Proteobacteria and Firmicutes, the most abundant phyla of the rock‐attached community included Actinobacteria (Figure 4a), which is in line with the most abundant phyla detected in borehole water by Boylan et al. (2019), while Bleyen et al. (2021) identified Chloroflexi as the third abundant phylum in borehole water. In the study by Boylan et al. (2019), the borehole community was dominated by only a few bacterial families such as Thermoanaerobacterales, Anaerolineaceae, and Desulfobulbaceae, which were not found in our study or were only detected at low abundances. In general, borehole water communities were much less diverse (Bagnoud, de Bruijn, et al., 2016; Boylan et al., 2019) than the rock‐attached community of this study and they were usually dominated by SRB. At only a few sample sites in the pristine OPA rock, we detected *Desulfosporosinus* and Peptocaccaceae, which were reported by Bagnoud, de Bruijn, et al. (2016) to be dominant in borehole water, but at very low abundances (Figure 5c). Only *Pseudomonas* was detected in all our samples at significant abundances. Contrary to the studies by Mauclaire et al. (2007) and Bagnoud, Chourey, et al. (2016) who focused on the cultivation of SRB and analyzed the borehole water, we did not detect *Desulfobulbus*, *Desulfocapsa*, and *Desulfotomaculum*. Our results are in line with the observation that rock‐attached communities differ in biodiversity and metabolic potential from suspended communities (Lehman et al., 2004). Reporting only the diversity of planktonic cells within a site is a commonly accepted practice, but it fails to capture significant differences between the distinct population of planktonic and attached cells in the deep subsurface (Lehman et al., 2004; Moser et al., 2003; Wanger et al., 2006). Although microorganisms will be more active at sites where they have space and water to grow, such as fractures and faults represented by boreholes in the current studies (Bagnoud, de Bruijn, et al., 2016; Bagnoud, Chourey, et al., 2016; Bleyen et al., 2021), our study provides valuable insights into expected microbial mechanisms of the rock‐attached communities in a repository. Solely studying fluids misses a potentially significant contribution from communities attached to surfaces as biofilms, especially considering that biofilm biomass represents 80% of all continental deep subsurface biomass (Flemming & Wuertz, 2019).

### 4.2 The importance of mineral‐associated microbial communities for safety purposes

In this study, we found numerous phylotypes or microbial traits with significant abundances which could be involved in processes relevant to the safety of a repository in OPA, such as corrosive biofilm formation, MIC as well as in mineral dissolution and/or transformation. In the following, these processes will be discussed based on the potential availability of electron donors/acceptors and the local mineralogy.

Corrosive biofilms are usually dominated by members of the phyla Proteobacteria, Firmicutes, and Bacteroidota (Procópio, 2020), which are the dominant bacterial phyla found in OPA rock (Figure 4a) (Procópio, 2020). Representative corrosion‐related orders such as Rhodobacterales and Sphingomonadales (Alphaproteobacteria), Burkholderiales (Gammaproteobacteria), Bacillales (Firmicutes), as well as Flavobacteriales, Bacteroidales, and Sphingobacteriales (Bacteroidota), occurred at high abundances in OPA (Figure A5)*. Sphingomonas* spp., ubiquitously present in OPA (0.04%–3.9%), can be involved in MIC of copper (White et al., 1996) and may thus be critical when copper canisters are being used to store radioactive waste. Furthermore, they can produce gellan‐related exopolysaccharides which have a high stability to high temperatures, salt concentrations, and a wide pH range (Vu et al., 2009; White et al., 1996). Hence, they are further important in biofilm formation which may protect cells from the heat generated by nuclear waste or from other adverse conditions. The most abundant bacterial classes in OPA, Alpha‐ and Gammaproteobacteria are often detected alongside *Bacillus* species during the initial stages of biofilm formation. Bacteroidota representatives, on the other hand, are primarily associated with the formation and maintenance of biofilm structures (Procópio, 2020). Although no biofilm formation has been observed in situ on excavated claystone, the disturbed host rock and clay buffer will inevitably be saturated with water after the repository closure enabling the formation of biofilms at least in interfacial areas (Meleshyn, 2014).

High abundances of the potentially biofilm‐forming phyla Proteobacteria and Bacteroidota were associated with high contents of the clay mineral illite (Figure 4a,c). Because mineral particles are composed of inorganic nutrients which can potentially be used by microorganisms, microbial communities can differ depending on the type of mineral and its inclusions (Casar et al., 2020; Uroz et al., 2015). Microorganisms can also play an important role in the genesis of minerals such as illite. Proteobacteria, for instance, were reported to be among the dominant phyla related to ceramic materials made of clay minerals including illite (Fomina & Skorochod, 2020).

Especially the iron content of minerals is known to affect the biomass as well as the structure and composition of microbial communities (Mitchell et al., 2013). Minerals with higher iron and phosphorus contents are preferentially colonized by bacteria (Phillips‐Lander et al., 2013). Similar to granitic rocks, the comparatively iron‐poor and poorly weatherable minerals quartz and plagioclase were the major geochemical driver of the microbial community structure in our study (Figure 3) (Ennis et al., 2021). Both minerals mainly occurred in the PW formation (Figure 2). At the same time, we observed a comparatively low proportion of bacteria compared to archaea within the PW formation.

The high contents of quartz and plagioclase (Figures 2b and 4) coincided with the occurrence of a methane‐cycling community (Figure 5). Methanogenic archaea were abundant (15%) and diverse. They included *Methanobacterium*, *Methanosarcina*, and Methanomassiliicoccaceae which are among the dominant archaea usually related to corrosion (Procópio, 2020). Metal corrosion mediated by methanogenic archaea can occur concomitantly with methane production (Larsen et al., 2010; Usher et al., 2014), but may be of concern only when microbial communities can overcome the technical bentonite barriers, that is, when disruptive events introduce them to the bentonite‐container interfaces. In addition to the contents of quartz and plagioclase, the lowest water and TOC contents as well as lower iron and sulfate concentrations in the PW formation may have created favorable conditions for hydrogenotrophic methanogenesis. In general, the overall TOC and water content were factors shaping the microbial community structure in OPA and the PW formation (Figure 3). A correlation between the microbial community and TOC was also observed by Wouters et al. (2013) in water samples from the underground Boom Clay facility in Belgium. The carbon content of OPA determined in the present study ranged between 0.8% and 1.1% (wt/wt) and is thus in the range of up to 1.5% (wt/wt) organic matter found in OPA (Pearson et al., 2003) and typical for deep clay formations (Meleshyn, 2014). Microbial processes such as fermentation and indirectly organotrophic methanogenesis rely on the presence of organic matter and are of high importance in the deep subsurface (Krumholz et al., 1997; Wersin et al., 2011). In accordance with the lowest TOC content (0.5%), the most abundant methanogen in the PW formation was *Methanobacterium* (9% of the total community), which is known for chemolithoautotrophic methanogenesis using solely CO_2_ and H_2_. Hydrogen can be produced abiotically through water–rock interactions or biotically through fermentation processes (Kietäväinen, 2015). Fermentative *Clostridia* were, however, less abundant in the PW formation (0.4%) and negatively correlated with quartz and plagioclase. The carbonate mineral calcite could serve as an inorganic carbon source. Carbonate dissolution can result in the localized formation of CO_2_ from CO_3_^2−^(Garcia‐Pichel et al., 2010; Guida & Garcia‐Pichel, 2016). In addition to methanogenic archaea, we found a high relative abundance of *Verrumicrobiales* such as *Luteolibacter* (21%), which are supposedly associated with methane oxidation (Gründger et al., 2021). Methane cycling communities in the deep subsurface have been recently described also for crystalline rocks (Kietäväinen, 2015).

Besides methanogenic archaea, other functional traits including SRB/sulfate‐reducing archaea and IRB/iron‐oxidizing bacteria are among the species commonly associated with metal corrosion (Beech et al., 2014). Following the availability of key terminal electron acceptors and donors in OPA, methanogenic organisms and SRB are expected to be outcompeted by IRB, inhibiting methane production and/or sulfate reduction in OPA (Chapelle, 2000). Our study shows that both OPA facies were characterized by only a small proportion of methanogenic archaea (<2%; Figure 5d). In general and contrary to the PW community, the OPA community was mainly composed of bacteria (Table A1).

Although the presence and activity of SRB in OPA borehole water were described in several studies (Stroes‐Gascoyne et al., 2007; Bagnoud, Chourey, et al., 2016; Bagnoud, de Bruijn, et al., 2016; Boylan et al., 2019), they were absent from the rock‐attached microbial community or only found at low abundances. Our results indicate that SRB represents only a minority of the rock‐attached microbial community in unperturbed OPA. Together with slow corrosion rates under anoxic conditions, our results suggest the insignificant potential for corrosion in unperturbed OPA. However, the potential impact of SRB should not be underestimated based on the comparatively low biomass, since their activity over geological time must be considered.

Like in many deep subsurface environments, Fe(III) could be the dominant electron acceptor for microbial respiration also in OPA (Lovley & Chapelle, 1995). We observed that OPA harbored a high diversity and abundance of potential IRB (4.3%–10.2%) dominated by *Pseudomonas* and *Acinetobacter* (Figure 5b). A wide variety of facultatively or obligately anaerobic microbial species have been found to reduce structural Fe(III) in clay minerals (Dong et al., 2009). *Pseudomonas* spp. are among the species that can reduce Fe(III) in clays (Gates et al., 1993) as well as facilitate the corrosion of iron and its alloys (Beech et al., 2014). *Acinetobacter* was found to be involved in iron reduction in sediments (Pan et al., 2017). Other potential IRB that have been associated with corrosion, such as *Sulfurospirillum*, *Desulfovibrio*, *Bacillus*, *Clostridium*, and *Acidiphilium* (Herrera & Videla, 2009), were observed at lower abundances (<1.6%) in OPA.

The reduction and dissolution of structural iron present in clay minerals such as illite, illite–smectite, kaolin, and chlorite could affect the integrity of insulating clays in a repository such as a bentonite barrier or the OPA host rock. The dominant occurrence of potential IRB supports a previous assumption that iron reduction could be the most safety‐relevant process in a DGR (Meleshyn, 2014). On the other hand, Fe(III) can act as an electron acceptor for the oxidation of H_2_ so that microbial Fe(III) reduction could reduce the risk of overpressure and corrosion processes related to abiotically formed H_2_ in a potential repository.

**Figure A6:**
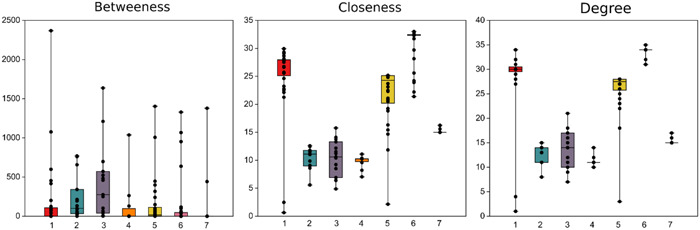
Network metrics for each of the Modules 1–7. Module 1, shaly‐3; Module 2, sandy‐3; Module 3, shaly‐1; Module 4, sandy‐2; Module 5, sandy‐1; Module 6, shaly‐2; and Module 7, PW.

**Figure A7:**
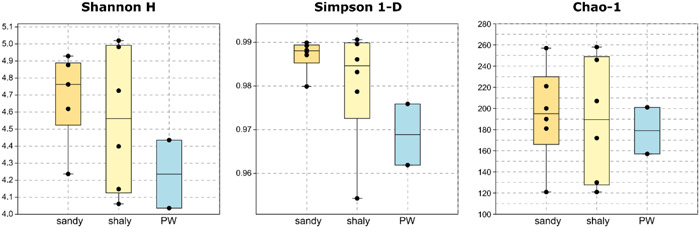
Alpha diversity indices in the sandy and shaly Opalinus Clay facies as well as in the Passwang formation.

The similarity of microbial communities from both OPA facies and the strong difference to the PW microbial community indicates the existence of a clay‐specific community that is driven by mineral composition. However, even slight differences in the mineral composition such as a higher amount of quartz in the OPA sandy facies or a higher concentration of calcite in the OPA shaly facies (Figure 2b) foster a formation of facies‐specific communities within the clay‐associated microbial community (Figure 3a). Dependence on the mineral and elemental composition of microbial assemblages is further evidenced by the network analysis which resulted in the formation of modules of co‐occurring microbial taxa in accordance with the sample location and its site‐specific mineralogical and geochemical characteristics (Figure 6). In three of the modules (OPA sandy‐1, OPA shaly‐2, and ‐3), the members were highly connected, and clustering appeared to be very dense (Figure A6). A correlation of the module OPA sandy‐1 with high pyrite and orthoclase as well as Fe and S contents (Figure 6c) suggested the presence of a local iron‐cycling community. Indeed, one of the abundant ASVs (>2%) found among the co‐occurring taxa was affiliated with *Segetibacter* (ASV_250; Supporting Information: Data S3), which was shown to be involved in the nitrate‐dependent anaerobic Fe(II)‐oxidation (Wang et al., 2019). Although, we could not measure nitrate in the leachates, nitrate and ammonium were previously detected in OPA borehole water (Pearson et al., 2003). Crystalline Fe(II)‐minerals, including pyrite, can be subject to direct nitrate‐dependent microbial oxidation (Weber et al., 2006), leading to the precipitation of a variety of Fe(III) minerals and/or mixed‐valent Fe(II)–Fe(III) mineral phases in anoxic settings (Bryce et al., 2018). Fe(III)‐minerals can, in turn, be subject to iron‐reducing processes mediated by potential IRB such as *Acinetobacter*, *Bryobacter*, and *Pseudomonas* found to be abundant at this site (Figure 5b) and within module OPA sandy‐1 of the co‐occurrence network (Supporting Information: Data S3).

High abundances of carbon‐fixing Planctomycetes and Proteobacteria within module OPA‐shaly‐2, an enrichment of predicted carbon fixation pathways (Figure A4) and an association of this module with calcite and, thus, with CaCO_3_, Ca, CO_2_, TC, and TIC (Figure 6c) suggest carbon fixation be an important process at this site. The most abundant carbon fixation pathway predicted here, the Calvin–Benson–Basham cycle (Figure A4), is a widely distributed pathway to fix CO_2_ by chemolithotrophs (Zhao et al., 2020). The main carbon source could be the local carbonate mineral calcite.

The co‐occurrence of organisms and their association with the site‐specific mineralogy may be an indication of metabolically active subcommunities within OPA. Similar observations of functional adaptation and community structuring by geological and hydrogeochemical conditions were made in deep continental crystalline rocks (Nyyssönen et al., 2014). Potentially iron‐reducing taxa (*Acinetobacter*, *Pseudomonas*, and *Bryobacter*) and all potentially corrosive biofilm‐forming orders were common not only in the overall communities but also in the site‐specific subcommunities (Supporting Information: Data S3).

The local‐scale variation in community composition is likely to result from the selection of certain organisms from a source community due to differences in mineralogical and geochemical conditions in each microhabitat. In addition to homogeneous selection, Whitman et al. (2018) described dispersal limitation to be important in controlling community assembly on mineral surfaces. In claystone environments, dispersal limitation is caused by low water activity and small pore space. Especially the association of the community structure with the water and TOC content as well as the subcommunity formation according to local geological conditions suggests an active postdepositional microbial community assembly and evolution, presumably over geological time scales.

Despite the described indication for potential microbial activity, the extracted DNA may originate from dormant cells or represent preserved extracellular DNA adsorbed to clay minerals (Franchi et al., 1999). To draw conclusions about actual activity, further methods such as examination of gene expression, protein synthesis, or metabolites must be undertaken. Nevertheless, this study provides a fundamental basis for describing the microbial communities and their relation to mineral composition in a potential host rock for DGRs.

## 5 CONCLUSIONS

The present study aimed at providing a scientific basis for evaluating the possible impact of OPA rock‐attached microbial communities on the safety of a final repository for HLW in clay‐rich host rocks. Amplicon‐based microbial community analysis revealed that OPA is dominated by bacterial phyla known to be involved in corrosive biofilm formation. Based on the abundance of corresponding microbial traits and available electron donors and acceptors, iron reduction appears to be the most important process, predominating over methanogenesis and sulfate reduction. Generally, OPA microbial communities are homogenous, and their composition strongly differs from the adjacent PW formation suggesting the presence of a claystone‐specific microbial community. Site‐specific mineralogical and related geochemical conditions have further selected for subcommunities and specific metabolic functions. These results suggest the presence of low‐active mineral‐associated microbial subcommunities and the potential for corrosion as well as mineral dissolution and/or transformation. This can pose risks to the safety‐relevant properties of a final repository if barriers suppressing microbial activity are weakened. Although, with our approaches, we cannot finally prove that microbial communities are active under in situ conditions, the study advances the current understanding of microbial life in the deep subsurface in general and provides a valuable contribution for evaluating safety‐relevant processes in a final repository in OPA. Future studies using metagenomic and transcriptomic approaches are needed to explore the active microbial communities and metabolic processes, but these techniques are challenging in low‐biomass environments. Knowledge of mineral‐associated microbial communities could be implemented in predictions of microbial communities and their functions in other potential host rock formations. However, studying the environmental conditions such as redox potential and availability of substrates together with microbiology at the particular repository site is crucial to understand metabolic cycles and the specific role of microorganisms.

## AUTHOR CONTRIBUTIONS

**Julia Mitzscherling**: Conceptualization (equal); formal analysis (equal); methodology (equal); visualization (lead); writing—original draft (lead); writing—review and editing (equal). **Steffi Genderjahn**: Formal analysis (equal); investigation (equal); methodology (equal); writing—review and editing (equal). **Anja M. Schleicher**: Conceptualization (equal); investigation (equal); methodology (equal); writing—review and editing (equal). **Alexander Bartholomäus**: Data curation (equal); methodology (equal); visualization (equal); writing—review and editing (equal). **Jens Kallmeyer**: Formal analysis (equal); methodology (equal). **Dirk Wagner**: Conceptualization (equal); funding acquisition (equal); supervision (equal); writing—review and editing (equal).

## CONFLICT OF INTEREST STATEMENT

The authors declare no conflict of interest.

## ETHICS STATEMENT

None required.

## Supporting information


**Data S1**. Geochemical and mineralogical parameters of samples used for microbial community analysis. i–s, illite–smectite mixed layers; calcite, including siderite & ankerite; LOI, loss of ignition.

**Data S2**. Genus‐level microbial community composition and their relative abundance.

**Data S3**. The abundance of co‐occurring ASVs in each rock sample representing one module. ASVs with > 2% abundance are highlighted in red. Indicator species of the Passwang Formation are highlighted in orange in module 7.

**Data S4**. Read counts (>20) and taxonomy of ASVs found in the negative controls including a blank kit extraction, blank PCR, and DNA extracts from N2 gas of the anaerobic chamber used for sample preparation. The table only includes ASVs that were filtered from the sample's ASVs.

## Data Availability

Sequencing raw data are publicly available via the European Nucleotide Archive under the Project accession number PRJEB53941: https://www.ebi.ac.uk/ena/browser/view/PRJEB53941.

## ADDITIONAL RESULTS

### 1

**Table A1:** Relative abundance of bacteria and archaea in each sample in %.

	sandy‐1		sandy‐2		sandy‐3		shaly‐1		shaly‐2		shaly‐3		PW	
	BMA3_1a	BMA3_1b	BMA3_2a	BMA3_2b	BMA3_3a	BMA3_3b	BMA4_1a	BMA4_1b	BMA4_2a	BMA4_2b	BMA4_3a	BMA4_3b	BMA3_4a	BMA3_4b
Archaea	6.27	7.06	0.84	1.12	1.34	3.59	0.57	2.03	1.91	3.04	3.69	0.29	39.77	21.71
Bacteria	93.73	92.94	99.16	98.88	98.66	96.41	99.43	97.97	98.09	96.96	96.31	99.71	60.23	78.29

Abbreviation: PW, Passwang formation.

**Table A2:** Indicator ASVs identified in the PW and their respective relative abundance in %.

Name	Group	Indicator value	*p*‐Value	sandy‐1	sandy‐2	sandy‐3	shaly‐1	shaly‐2	shaly‐3	PW	Phylum	Class	Order	Family	Genus
ASV_.128	PW	1	*0.026*	0	0	0	0	0	0	3.36	Crenarchaeota	Bathyarchaeia	NA	NA	NA
ASV_.171	PW	1	*0.014*	0	0	0	0	0	0	2.53	Crenarchaeota	Bathyarchaeia	NA	NA	NA
ASV_.302	PW	1	*0.014*	0	0	0	0	0	0	1.38	Crenarchaeota	Nitrososphaeria	Nitrososphaerales	Nitrososphaeraceae	NA
ASV_.69	PW	1	*0.012*	0	0	0	0	0	0	5.87	Euryarchaeota	Methanobacteria	Methanobacteriales	Methanobacteriaceae	*Methanobacterium*
ASV_.138	PW	1	*0.013*	0	0	0	0	0	0	3.25	Halobacterota	Methanosarcinia	Methanosarciniales	Methanosaetaceae	*Methanosaeta*
ASV_.233	PW	1	*0.012*	0.05	0	0	0	0	0	1.61	Actinobacteriota	Actinobacteria	Propionibacteriales	Nocardioidaceae	*Nocardioides*
ASV_.229	PW	0.96	*0.016*	0	0	0	0.15	0	0	1.37	Proteobacteria	Gammaproteobacteria	Burkholderiales	Comamonadaceae	*Rhodoferax*
ASV_.36	PW	0.99	*0.008*	0	0	0	0.30	0.15	0	11.92	Verrucomicrobiota	Verrucomicrobiae	Verrucomicrobiales	Rubritaleaceae	*Luteolibacter*
ASV_.67	PW	1	*0.009*	0	0	0	0	0	0	5.71	Verrucomicrobiota	Verrucomicrobiae	Verrucomicrobiales	Rubritaleaceae	*Luteolibacter*
ASV_.189	PW	1	*0.02*	0	0	0	0	0	0	2.32	Verrucomicrobiota	Verrucomicrobiae	Verrucomicrobiales	Verrucomicrobiaceae	NA
				**0.05**	**0.00**	**0.00**	**0.45**	**0.15**	**0.00**	**39.32**					

Abbreviations: ASV, amplicon sequence variant; PW, Passwang formation.

Members of the modules Opalinus Clay (OPA) shaly‐3 (Module 1), OPA sandy‐1 (Module 5), and OPA shaly‐2 (Module 6) possessed the highest degree of centrality meaning that most members in those clusters were highly connected with others and had a high level of co‐occurrence within that cluster. With regard to the closeness, similar observations were made highlighting the dense clustering of nodes in those modules. The betweenness of the respective modules was lower as of the modules OPA sandy‐3 and OPA shaly‐1, in which the degree was medium‐high, and the closeness was lowest. However, one single node of module OPA shaly‐3 possessed the highest betweenness of all modules. Members of a network that have both high degree and betweenness centrality are the most connected taxa and are considered “hubs” that may have an ecological relevance to the community (1). The highest hub scores were observed in module OPA shaly‐2 possessing the maximum degree and closeness centrality. Members of module PW were observed to have low a degree and closeness centrality, the minimum betweenness centrality, and thus hub scores. Minimum values of the betweenness centrality were a result of the divergence from and low connectivity with members of other modules.
